# How to organize science and technology information in Latin America?

**Published:** 2016-09-30

**Authors:** Mauricio Palacios

**Affiliations:** Editor Asociado, Revista Colombia Médica. Universidad del Valle, Cali, Colombia

To better guide science and technology policies, it is required high quality and updated information on organizations, researchers, projects and products. The growth of the Internet use in research has provided more information on these aspects; however, the volume of data made more difficult the methods for processing and organizing them in a way useful to understand and make informed decisions. Problems such as duplication of information, difficulties in monitoring processes (authors and projects with products), and the lack of identification of thematic research and knowledge networks have increased in the last twenty years. In all this, the most important factor hindering the organization of data has been the need to identify each component.

Perhaps the most complicated element of the ecosystem of science to be identified is the researchers, because there is a lot of variability in the way of writing the names, the institutional affiliation and the contact email. Additionally, homonyms, typos and citation mistakes significantly affect any database 1,2 . For this reason, many proposals to identify persons have been generated in the world since the 1990s. Latin America also launched an identification proposal of the researchers called *Proyecto del Currículum Vitae en Ciencia y Tecnología de América Latina y el Caribe*, better known as CVLAc. This project adopted the Curriculum Lattes technology and methodology developed by the *Consejo Nacional de Desarrollo Científico y Tecnológico de Brasil (CNPq)*. It was ambitious in its objectives, with open access to share information, and it was implemented with the support of the Pan American Health Organization (PAHO) and the *Centro Regional de Información de Ciencias de la Salud (BIREME)* at the beginning of this century 3. CVLAc did not achieve all its objectives; although it has been the identification system in Latin America that has called for more researchers, and on which it was supported the seeking of peer reviewers. In addition, it inspired other developments related to research work in the region, such as databases of groups and journals. The tool was successful in the first decade of this century.

Currently, CVLAc presents several problems arising from a limited development posterior to its implementation. The most prominent one is the isolation from other international identification systems. Without developments in the acquisition of metadata, information integration and harmonization of identifiers, the interest is limited to a reciprocal initiative with some of the systems with more coverage, such as Researcher ID, Research Gate, Google Scholar, or even from the region as the *Autores Redalyc*. The second problem is transparency in information. CVLAc doesn't have a validation system on data entry to detect errors or duplication of information. It is still performed a manual processes for verification by calling, and this has enabled some cases of questionable conduct. There are other aspects (manual update, data storage, etc.) that detract attractiveness and competitiveness compared to other databases. For these reasons it can be considered that CVLAc and the developments derived from it do not have quality information to support policies and actions intended to the scientific development of a country. 

These difficulties with information systems in science occur in varying degrees in many countries 4, and alternative solutions must have the infrastructure of interoperability to provide consultation with three attributes: Identification, co-reference and semantics 5. Information management research requires open access programs, to avoid over costs to institutions or researchers; with standardized semantics to link to each other, information from several databases; with systems of universal identification, to avoid duplication errors and fragmentation of information; and with open code, to make the necessary changes required by the particular forms of countries or regions in the way of doing research, associating among themselves and publishing 6. With these requirements, there are highlighted some programs that are the base of information in countries with research leadership.

 The Digital Object Identifier system (DOI) implemented and supported by DataCite has been the most clear advance, and with measurable results after its implementation. In the case of journal articles, book chapters and other documents resulting from research, the marking is standardized, and the semantics is performed with a controlled language. In practice, the use of DOI avoids duplication of products, and it facilitates bibliometric analysis, and the interoperability with other databases is performed without difficulties 7. The implementation is institutional or editorial, with low costs.

Inspired by the DOI, it was developed the researchers' identification system ORCID (Open Researcher and Contributor ID). It is an international open access system created and supported by Consortia Advancing Standards in Research Administration Information. ORCID has interoperability with other identification systems, such as Researcher ID and *Autores Redalyc* (for authors in Latin American magazines). They can import information of products where the author uses his/her ORCID identification, and it has audits on the application of a product identified with the DOI by more than a homonym author. It is recognized in the world because it is the system that most contributes to the disambiguation of authors, and it improves transparency in the way of publishing. Some countries have adopted it as an official initiative for their information systems, including Australia and Italy 8. In Italy, the implementation of ORCID achieved a linkage of over 80% of researchers in the first 6 months, and improved information flow on research centers and the state agency for science and technology 9.

Institutionally, it is required an information manager that interacts with the two previous systems. So far, DSpace software, developed by the Massachusetts Institute Technologic, has shown the best capacity to adapt to the particularities of each research center or region. Widespread adoption for a country does not depend on a government initiative. The Organization of Italian Universities, ANDU, defined its use as an academic enterprise, which was later endorsed by the Italian Ministry of Education and Research 9. For research institutions, it would partly solve the invisibility of Latin American science 10.

A little less defined, because of the complexity of the task and the volume of information to process, are the national information systems on science and research. At this level, there is no consensus on the program to use; but the developments can be analyzed from the integration of the institutional DSpace systems 11.

All these systems require the willingness of researchers, as well as the science and technology government institutions and organizations. But convincing each one of the participants can be easier because these systems remunerate participation in forms of thematic consultations, by processes, bibliometric, among others. Discussions during the implementation on information persistence issues could be generated, and on authority and confidence; valid and necessary for society; but that do not contradict their usefulness for research systems.

With appreciation for the contribution made by the CVLAc and its derived databases, it is time to abandon them; we are in a time for Latin America having an increased diffusion and dissemination of its science, and critical thinking to the world with universal identity and languages. 
